# Comparing clinical outcomes of Optiwave Refractive Analysis, Lenstar, and surgeon’s modified method for intraocular lens power calculation in Asian eyes

**DOI:** 10.1038/s41598-023-41720-2

**Published:** 2023-09-02

**Authors:** Hung-Yuan Lin, Shu-Ting Kao, Shuan Chen, Ya-Jung Chuang, Pi-Jung Lin

**Affiliations:** 1 Universal Eye Center, Zhong-Li, Taipei City, 10660 Taiwan; 2 Department of Optometry, Central Taiwan University of Science and Technology, Taichung, 40601 Taiwan; 3 Department of Ophthalmology, Fujian Medical University, Fuzhou city, 350005 Fujian Sheng China; 4 Yee-Hong Clinic, New Taipei City, 23447 Taiwan; 5 Universal Eye Center, Long-Tan, Taipei City, 10660 Taiwan

**Keywords:** Biomarkers, Predictive markers

## Abstract

The study aimed to compare the accuracy of intraocular lens (IOL) calculation to predict postoperative refraction by Optiwave Refractive Analysis (ORA), Lenstar LS 900, and the surgeon’s Modify method in normal Asian eyes. The IOL power of the Lenstar group was calculated according to Lenstar LS 900, whereas the surgeon's Modify group used topography, axial length (AL) of Lenstar, and Barrett Universal II online formula. Intraoperative aphakic measurements and IOL power calculations were obtained with the ORA system. From the results acquired through Lenstar, Modify, and ORA, the surgeon used his judgment to select the actual IOL power. Postoperative manifest refraction spherical equivalent (MRSE) was obtained 2 months after surgery. The prediction error (PE) was calculated as the difference between the postoperative MRSE and the target refraction proposed by three methods. AL, anterior chamber depth (ACD, measured from corneal endothelium to lens), lens thickness (LT), and ACD + 1/2LT were also included in the survey. In 67 eyes, the average real PE was smaller for the Lenstar (0.06 ± 0.44) and Modify (− 0.05 ± 0.40) than for the ORA group (− 0.25 ± 0.60, *p* < 0.05). The ORA system demonstrated the best results of IOL power selection in eyes with a normal range of ACD + 0.5 LT (5.2–5.6 mm) in Asian eyes.

## Introduction

Nowadays, cataract surgery has remarkably advanced. The success and safety of this procedure are attributable to continuous improvement in surgical technique and measurement methods. At the same time, patients have high expectations for visual outcomes, and spectacle independence has made precise refractive targets an increasingly important component of cataract surgery^1,2^. However, minimum targets for refractive outcomes in virgin eyes as expectations proposed by the National Health Service (NHS) of the United Kingdom, which currently represent 55% within 0.5 D and 85% within 1 D of emmetropia, have not been yet achieved in every case^3^. In a recent large study including more than 11,083 cases for the last 10 years, the spherical equivalent of 7938 eyes (88.76%) was within 1 D, and that of 5577 eyes (62.36%) was within 0.50 D after cataract surgery^1^. Achieving the predicted postoperative spherical equivalent (SE) remains a major concern in cataract surgery, although modern optical biometry and surgical technology have advanced.

Intraocular lens (IOL) calculation accuracy of the conventional methods usually involving several factors to achieve postoperative emmetropia includes the surgeon factor, axial length (AL), biometry measurements, and additional measurements in some formulas, e.g., anterior chamber depth (ACD, measured from corneal endothelium to lens) and lens thickness (LT). However, the real-time intraoperative aberrometry (IA) during cataract surgery, Optiwave Refractive Analysis (ORA) (Alcon, Fort Worth, TX, USA) system estimating IOL power based pura ely on refractive algorithm without AL and keratometry measurements during cataract surgery in an aphakic state, could transcend this uncertainty^4–6^. Thereby, the refractive outcome may be improved, especially in complicated cases, such as those after refractive surgery^7,8^. Yet, a considerable debate exists about the reliability of intraoperative wavefront aberrometry in producing stable results in normal eyes^9–11^.

This study aimed to evaluate the accuracy of intraoperative wavefront aberrometry for IOL power selection in virgin eyes compared to Lenstar LS900 (low coherence reflectometry with integrated Barrett formulas, Haag-Streit AG, Koeniz, Switzerland) and Modify method (placido disk-based topography platform, Topolyzer, WaveLight, Alcon with online Barrett formulas II) in normal eyes. To the best of our knowledge, this is the first study assessing the effectiveness of the ORA system performed during cataract surgery in normal Asian eyes.

## Results

A total of 67 eyes of 53 patients was included between October 2019 and December 2020. The mean age was 67.1 (standard deviation = 8.3) years, and females were a predominant study population (n = 33, 62.3%) (Table 1).

**Table 1:** Basic characteristics of eyes and patients.

Variable	Number (%) or mean ± SD
Number of patients	53
Number of eyes	67
Age, year	67.1 ± 8.3
Female sex	33 (62.3)
Eyes	
OD	32 (47.8)
OS	35 (52.2)
IOL type	
CNA0T0	3 (5.7)
MF15	14 (26.4)
MF15T1	1 (1.9)
MF20	12 (22.6)
MX60	1 (1.9)
PCB00	1 (1.9)
SA60AT	13 (24.5)
SN6AT4	1 (1.9)
ZCB00	7 (13.2)
LENSTAR	
Keratometry 1 (D)	43.6 ± 1.6
Keratometry 2 (D)	44.4 ± 1.6
Axial length (mm)	23.9 ± 1.3
Anterior chamber depth (mm)	3.1 ± 0.3
Lens thickness (mm)	4.6 ± 0.3
Implanted IOL power (D)	19.5 ± 3.5
Predicted refraction in SE (D)	− 0.15 ± 0.27
ORA	
Keratometry 1 (D)	43.8 ± 1.6
Keratometry 2 (D)	44.4 ± 1.5
Implanted IOL power (D)	19.5 ± 3.5
Predicted refraction in SE (D)	− 0.46 ± 0.42
MODIFY	
Keratometry 1 (D)	43.5 ± 1.5
Keratometry 2 (D)	44.3 ± 1.6
Implanted IOL power (D)	19.5 ± 3.5
Predicted refraction in SE (D)	− 0.26 ± 0.30

*SD* standard deviation, *IOL* intraocular lens, *D* diopters, *SE* spherical equivalent.

### PEs of the target refraction

PE that the postoperative MRSE compared to the target refraction SE for the three proposed methods (Lenstar, ORA, and Modify) are shown in Table 2. The PE was expressed both in absolute error with only positive values and real error including both positive and negative values, referring to either more hyperopic or myopic results than targeted. The average real PE was relatively smaller for the Lenstar (mean ± SD: 0.06 ± 0.44) and Modify methods (− 0.05 ± 0.40 and relatively greater for the ORA method (− 0.25 ± 0.60, *p* = 0.023).

**Table 2:** Prediction error of LENSTAR, MODIFY and ORA target refraction and the postoperative manifest refraction.

Variable	Real prediction error (D)		Absolute prediction error (D)		Number (proportion) in error range (%)		
	Mean ± SD	Median (min, max)	Mean ± SD	Median (min, max)	≤ ± 0.5D	0.5 D-1.0 D	> ± 1.0D
MRSE	− 0.21 ± 0.46	− 0.25 (− 1.75, 0.88)	0.36 ± 0.35	0.25 (0.00, 1.75)	51 (76.1)	12 (17.9)	4 (6.0)
Difference							
MRSE and LENSTAR TRSE*	0.06 ± 0.44	0.04 (− 1.15, 1.14)	0.34 ± 0.29	0.27 (0.00, 1.15)	49 (73.1)	14 (20.9)	4 (6.0)
MRSE and MODIFY TRSE^#^	− 0.05 ± 0.40	− 0.05 (− 0.89, 1.17)	0.31 ± 0.25	0.23 (0.01, 1.17)	55 (82.1)	11 (16.4)	1 (1.5)
MRSE and ORA TRSE^&^	− 0.25 ± 0.60	− 0.31 (− 1.79, 1.18)	0.51 ± 0.39	0.41 (0.02, 1.79)	41 (61.2)	18 (26.9)	8 (11.9)

*D* diopters, *SD* standard deviation, *MRSE* manifest refraction spherical equivalent, *TRSE* target refraction spherical equivalent

*P value = 0.231 for differences of MRSE and Lenstar TRSE versus differences of MRSE and ORA TRSE;

^#^*P* value = 0.112 for differences of MRSE and Lenstar TRSE versus differences of MRSE and MODIFY TRSE;

^&^*P* value = 0.023 for differences of MRSE and ORA TRSE versus differences of MRSE and MODIFY TRSE.

Likewise, the absolute PE was relatively lower for the Lenstar (0.34 ± 0.29) and Modify methods (0.31 ± 0.25) and relatively higher for the ORA method (0.51 ± 0.39). There were 49 (73.1%), 55 (82.1%), and 41 (61.2%) eyes with small absolute errors (< 0.5 D) for the Lenstar, Modify, and ORA groups, respectively. On the other hand, there were 4 (6.0%), 1 (1.5%), and 8 (11.9%) eyes with large absolute errors (> 1 D) for the Lenstar, Modify, and ORA groups, respectively.

The difference in the absolute error classification between the ORA and Modify groups was statistically significant (*p* = *0.023*), while the difference between the Lenstar and ORA or between Lenstar and Modify groups was not significant (*p* = 0.231 and 0.112, respectively) (McNeMar test, Table 2).

**Figure 1:**
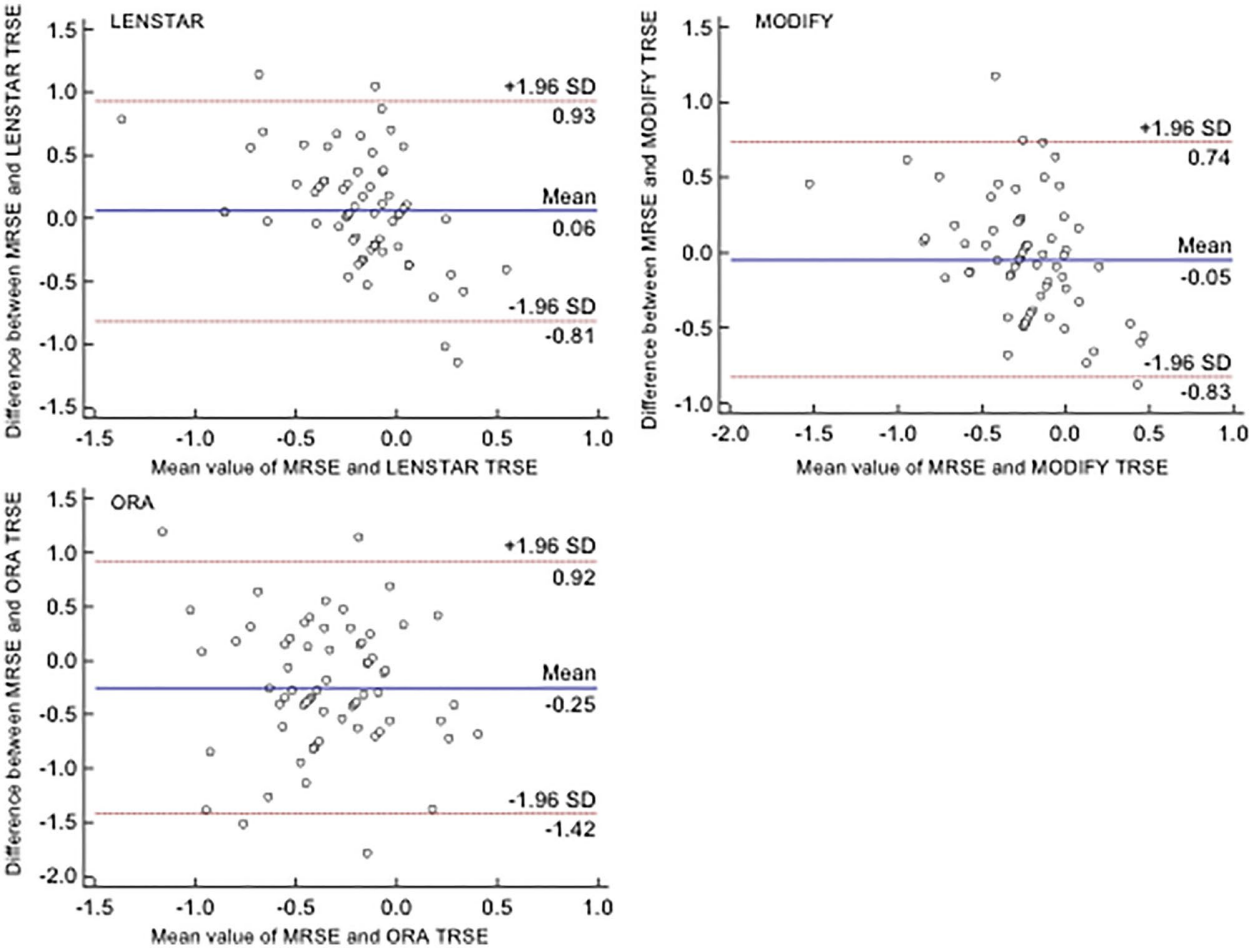
The Bland–Altman plots illustrated the agreement between target refraction and postoperative manifest refraction. Lenstar, Modify and ORA. *SD* standard deviation, *MRSE* manifest refraction spherical equivalent, *TRSE* target refraction spherical equivalent.

The agreement between each target refraction and MR analyzed by the Bland–Altman plots is shown in Fig. 1, illustrating that the width of the 95% confidence intervals (± 1.96 SD) of the difference in the PE was the widest (− 1.42 to 0.92) for the ORA method and was narrower for the Lenstar (− 0.81 to 0.93) and Modify methods (− 0.83 to 0.74) (Fig. 1).

#### PE of target refraction by different clinical characteristics

Table 3 demonstrates the difference in PE between MRSE and proposed methods by different clinical characteristics, including the subgroups of AL, ACD, LT, and the combination of ACD and LT (ACD + 0.5 × LT).

**Table 3:** The difference in the real prediction error between MRSE and proposed methods with different clinical characteristics.

Variable	Number of eyes	Difference between MRSE and LENSTAR TRSE (Mean ± SD, D)	*P*	Difference between MRSE and ORA TRSE (Mean ± SD, D)	*P*	Difference between MRSE and MODIFY TRSE (Mean ± SD, D)	*P*
AL			0.502		0.646		0.455
≤ 22 mm	18	0.01 ± 0.48		− 0.35 ± 0.63		− 0.14 ± 0.43	
23–24 mm	36	0.04 ± 0.42		− 0.23 ± 0.56		− 0.04 ± 0.39	
≥ 25 mm	13	0.19 ± 0.48		− 0.15 ± 0.67		0.05 ± 0.38	
ACD			0.284		0.154		0.068
≤ 2.9 mm	22	0.03 ± 0.50		− 0.19 ± 0.67		− 0.12 ± 0.38	
3.0–3.3 mm	32	0.14 ± 0.34		− 0.17 ± 0.52		0.07 ± 0.39	
≥ 3.4 mm	13	− 0.09 ± 0.55		− 0.54 ± 0.59		− 0.21 ± 0.41	
Lens thickness			0.196		0.334		0.454
≤ 4.3 mm	18	0.22 ± 0.43		− 0.14 ± 0.38		0.05 ± 0.38	
4.4–4.6 mm	27	− 0.02 ± 0.41		− 0.38 ± 0.74		− 0.07 ± 0.39	
≥ 4.7 mm	22	0.02 ± 0.48		− 0.18 ± 0.53		− 0.10 ± 0.43	
ACD + 0.5LT			0.012		0.010		0.009
≤ 5.1 mm	19	− 0.07 ± 0.50		− 0.34 ± 0.63		− 0.16 ± 0.40	
5.2–5.6 mm	37	0.20 ± 0.33^a^		− 0.08 ± 0.53^a^		0.08 ± 0.36^a^	
≥ 5.7 mm	11	− 0.18 ± 0.55^b^		− 0.67 ± 0.54^b^		− 0.27 ± 0.40^b^	

*MRSE* manifest refraction spherical equivalent, *TRSE* target refraction spherical equivalent, *SD* standard deviation, *D* diopters, *AL* axial length, *ACD* anterior chamber depth, *LT* lens thickness.

“a” and “b” indicate significant difference versus the “≤ 5.1 mm” and “5.2–5.6 mm” groups.

The results showed that the differences in PE have statistical significance in ACD + 0.5 LT for the three measurements (Lenstar, *p* = 0.012; ORA, *p* = 0.010; Modify, *p* = 0.009), while there were no significant differences among the subgroups of AL, ACD, and LT for the three measurements. Additionally, the difference in PE was significantly smaller in eyes with ACD + 0.5 × LT within 5.2–5.6 mm compared with other ranges in the three measurements. Moreover, a borderline significant trend that the difference between MRSE and Modify was smaller in the 32 eyes with normal ACD (3.0–3.3 mm, *p* = 0.068) was observed.

**Figure 2:**
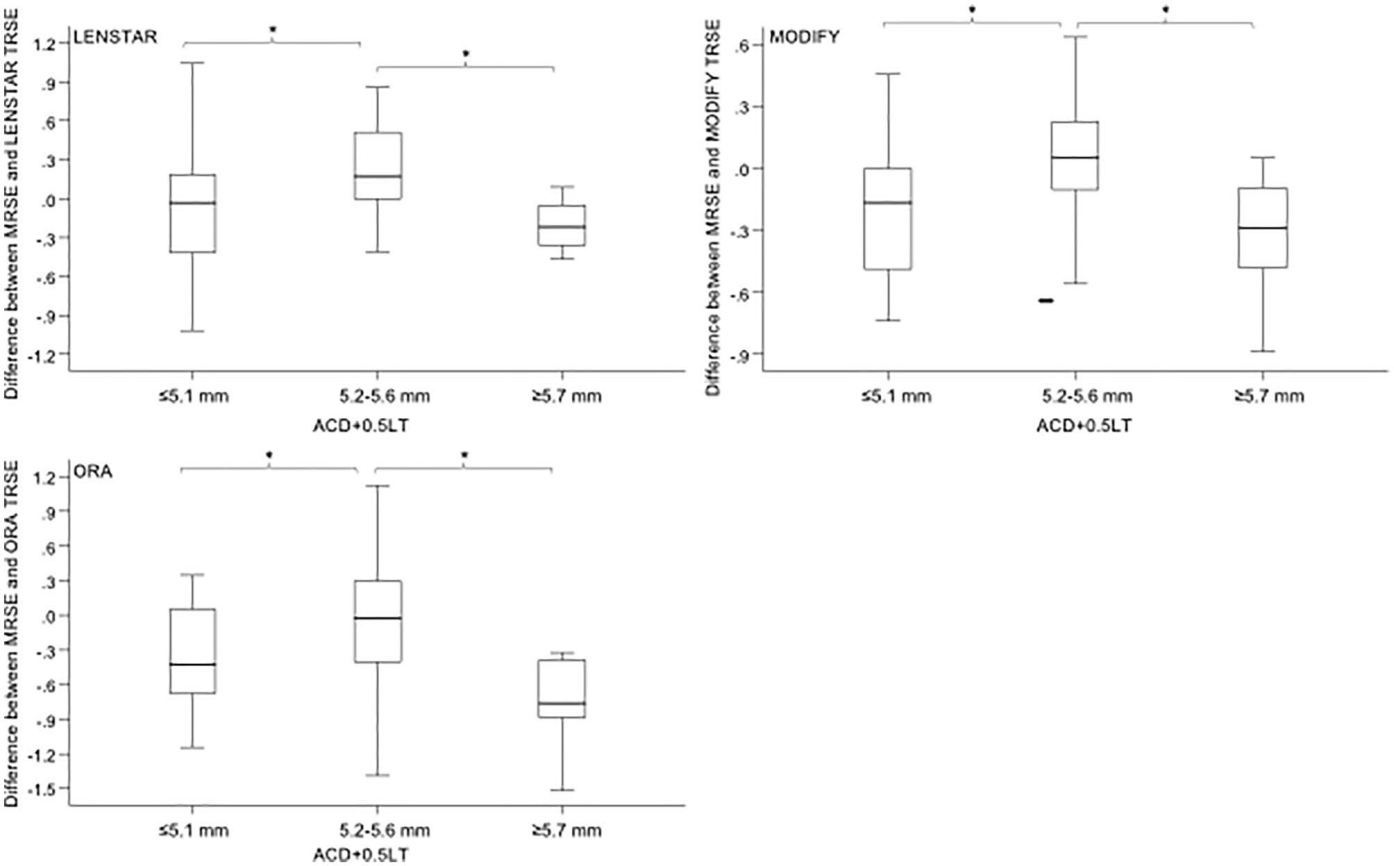
The prediction error of target refraction for the three proposed methods according to ACD + 0.5 × LT: Lenstar, Modify and ORA. *ACD* anterior chamber depth, *LT* lens thickness, *MRSE* manifest refraction spherical equivalent, *TRSE* target refraction spherical equivalent. The marker “*” indicates significant different at *P* < 0.05 level.

PE of target MR for the three proposed methods according to ACD + 0.5 × LT is shown in Fig. 2. The difference in PE was significantly smaller in the 37 eyes with normal ACD and LT (5.2–5.6 mm, *p* = 0.010) for each of the three measurements in ORA group.

### Correlation between the clinical characteristics and postoperative MRSE

Table 4 shows the correlation between preoperative clinical characteristics and postoperative MRSE in the second month. The results demonstrated that a longer AL was significantly correlated to a poor postoperative MRSE value (*r* =  − 0.28, *P* = 0.024). Additionally, a greater value of Modify-K1 was significantly correlated to a smaller postoperative MRSE (*r* =  − 0.22, *p* = 0.076). By contrast, there was no significant correlation between other factors and postoperative MRSE.

**Table 4:** The correlation between preoperative clinical characteristics and postoperative MRSE.

Variable	Pearson’s *r*	*P* value
LENSTAR Keratometry 1 (D)	− 0.16	0.190
LENSTAR Keratometry 2 (D)	− 0.12	0.315
AL (mm)	− 0.28	0.024
ACD (mm)	0.01	0.931
LT (mm)	0.11	0.385
ACD + 0.5LT (mm)	0.08	0.540
VERION K1 (D)	− 0.15	0.236
VERION K2 (D)	− 0.13	0.290
MODIFY Keratometry 1 (D)	− 0.22	0.076
MODIFY Keratometry 2 (D)	− 0.18	0.144

*MRSE* manifest refraction spherical equivalent, *D* diopters, *AL* axial length, *ACD* anterior chamber depth, *LT* lens thickness.

## Discussion

Refractive outcomes, especially spectacles independence after cataract surgery, play a critical role in shaping patient satisfaction. Although the advances in modern technology improve our ability to reach postoperative emmetropia, unexpected refractive errors still occur in many patients. Therefore, intraoperative wavefront aberrometry is one tool that may help improve IOL accuracy. It has shown to be helpful in prior refractive surgery cases^12^. However, several concerns about its precision and reliability need to be addressed. This study mainly aimed to evaluate the differences between the ORA PE and the conventional biometry PE with optical biometry and topography.

In our study, ORA provided a result within ± 0.5 D of the absolute PE 61.2% of the time, whereas the Lenstar and Modify calculations provided a result within ± 0.5 D of the absolute PE 73.1% and 82.1% of the time, respectively. The differences in MRSE and ORA TRSE vs. differences in MRSE and Modify TRSE have statistical significance (*P* = 0.023, McNeMar test), showing that Lenstar and Modify calculations could be superior to ORA calculation in cataract surgery in normal Taiwanese eye.

Regarding refractive outcome, generally, SE of < 0.5 D is within the target, and anything outside of that range is more likely to cause a disappointing outcome for a patient^13^. However, 11.9% of eyes in the ORA group showed large absolute errors (> 1 D), which could cause a patient to request an enhancement. Likewise, the results showed that ORA has a greater mean real PE than the other two methods. There were no restrictions on AL or IOL power in our study since the ORA system global optimized lens constant, and regression coefficients have been updated for each IOL model, not requiring exclusion of extreme AL. Our results demonstrated that greater AL was significantly correlated to a poor postoperative MRSE value (*r* =  − 0.28, *P* = 0.024), as consistent with previous results.

A limitation of the current study is the small sample size including only 67 normal eyes. Nevertheless, while this was limited to a retrospective single-center study with a single experienced surgeon, this also likely decreased any differences caused by the surgeon’s technique. Moreover, the differences in our study were small because these three calculations often provided very similar IOL power recommendations based on their target refractions. Yet, the results represent real-world outcomes, having a real-world value. In a large retrospective analysis of more than 13,000 normal eyes using a single optical biometer (Lenstar 900), Acrysof SN60WF IOL (Alcon Laboratories, Inc.), and the Barrett Universal II formula, Melles et al.^14^ have found a mean absolute error of 0.311 and 80.8% of eyes within ± 0.50 D of the target, which is close to our study. In our study, the mean absolute error of Lenstar was 0.34, and that of Modify was 0.31, while it was relatively higher for ORA (0.51). Besides, there is no significant difference in the K reading value in the Modify group and the Lenstar group, the IOL power calculation had no significant difference in these two groups.

Several published studies have reported conflicting results regarding the accuracy of IA vs. conventional formula with optical biometry. Cionni et al.^15^ have retrospectively analyzed 6460 eyes and concluded that IA produces more accurate SE outcomes for eyes implanted with a low toric IOL than the preoperative formula^13,15^. However, one of the arguments is that modern biometry devices and modern IOL formulas provide better refractive outcomes than the older technology used in Cionni's study^10,16^. Huelle et al.^9^ have argued that IA is not a reliable method for IOL calculation due to its high rate of measurement failures and large reading variations. Similarly, Davision et al.^16^ readings proposed that using IA to determine the IOL power in normal eyes does not improve overall expected clinical outcomes. However, it may be helpful in cases where the difference between IA and Preop calculations is high. Sudhakar et al.^17^ readings found that IA is not significantly different from the best preoperative biometry-based methods available for IOL power selection in short eyes. Certainly, the ORA does not replace surgeon’s selection but influences surgeons’ IOL selection and decision-making. In a retrospective study by Ianchulev et al.^12^, of the total 246 eyes, ORA either influenced (38%) or was chosen (30%) over the preoperative IOL power calculation in 68% of cases. When the powers of the ORA-recommended lens and the preoperatively planned lens differed, the ORA-recommended IOL power was used more often than the preoperatively planned lens.

Several factors may influence the accuracy of IA, including patient fixation, intraocular pressure, residual viscoelastic in the anterior chamber, increased corneal thickness, and external pressure from the lid speculum or squeezed eyelid.

Additionally, even if ORA measurements can be performed with high accuracy, the optical characteristics of an eye during surgery are slightly different from those of an eye under normal conditions. For instance, the postoperative IOL implant position within the eye, referred to as the ELP, plays an important part in refractive outcome prediction. ELP depends on several factors, including ACD, IOL thickness, IOL shape, and corneal power. However, the IOL position estimation based on the preoperative measurement is the largest source of uncertainty to choose an appropriate IOL power. Significant discrepancies between predicted and actual postoperative ELP result in refractive surprise^18^. Postoperatively, ELP cannot be directly measured. It would be measured by the ACD of the pseudophakia eye that can be measured by optical biometry or an optical coherence tomography (OCT) imaging device. Olsen et al. have found that 42% of IOL power PE was caused by incorrect postoperative ACD prediction^19^. Hence, modern IOL power calculation formulas, such as Holladay II, Olsen, Barrett Universal II, and Haigis, use preoperative ACD among the parameters to predict postoperative ELP.

However, some studies have reported that ACD value changes after cataract surgery. The postoperative ACD changes have been related to the preoperative ACD and AL, which impacted the refraction status and visual quality^20–22^. Both Ning et al. and Muzyka-Wozniak et al. have confirmed that the relative postoperative ACD changes were larger in short eyes (AL < 22.0 mm) than in normal or longer eyes (AL > 26.0 mm), meaning that the AL influenced changes.

Chui et al.^23^ have hypothesized that ELP on the distance from the corneal apex to the mid-sagittal plane of the cataractous lens would more accurately reflect the position of the principal plane of the non-angulated IOL within the capsular bag. They have found that the predictions of ELP with a preoperative value of ACD + ½LT could achieve greater accuracy and reliability than ACD alone. They have reported that accounting for half the value of LT in addition to ACD resulted in a significant reduction in mean ELP PE compared to using ACD alone, from − 1.57 ± 0.20 mm to 0.48 ± 0.16 mm.

In our study, we found that the differences in the PE between MRSE and proposed methods were significantly related to the combination of ACD and half LT (ACD + 0.5 LT), whereas there was no statistically significant difference among the subgroups of AL, ACD, and LT for the three measurements.

Furthermore, regarding the anatomical differences among ethics, Qin et al. have reported that Asian eyes had smaller anterior segments compared to Caucasian eyes measured by OCT^24^. Wang et al.^25^ have found that Caucasians had a significantly greater ACD, anterior chamber width, and corneal arc depth than all Chinese groups even after adjustment for refractive status and AL. Hence, this study provides first-hand information on ORA system application in cataract surgery in Asian eyes.

Although his study was limited by a small sample size and an absent control group, ORA system accuracy in IOL power selection demonstrated the same clinical outcomes as preoperative conventional biometry with Lenstar and surgeon's Modify method in Asian eyes. Further prospective larger studies and measurement modeling optimization need to be pursued to increase ORA system accuracy in the feature. Our results suggest applying the ORA system in IOL power selection in eyes with a normal range of ACD + 0.5L T (5.2–5.6 mm). The ORA can be used in patients within this range to obtain the best result.

## Methods

This was a nonrandomized, consecutive, retrospective, single-center study. All surgeries were performed by the same surgeon (H-Y Lin) at the Zhong-Li branch of Universal Eye Center. Approval for this study was obtained from the Institutional Review Board of the Antai Tian-Sheng Memorial Hospital, Taiwan, and the study was conducted in accordance with the tenets of the Declaration of Helsinki. Informed consent was waived by the Institutional Review Board of Antai Tian-Sheng Memorial Hospital due to retrospective nature of study.

### Inclusion and exclusion criteria

The inclusion criteria for participation were the age of > 50 years, no history of ocular trauma or surgery, no ocular surface disease, clear corneal media, attainable full pupil dilation (> 6 mm), and having postoperative manifest refraction (MR) at first- and second-months follow-up examinations. Exclusion criteria were corneal disease, poorly controlled diabetes (fasting sugar > 200 mg/dl), or any ocular pathology except for senile cataract. Eyes with unreliable preoperative biometric measurements, such as hypermature cataracts, also were excluded. Patients who met the enrollment criteria were included in the analysis regardless of the selected IOL type.

### IOL power selection

In addition to the ORA system IA, patients’ preoperative data were obtained by low coherence reflectometry Lenstar LS900 (Lenstar, Haag-Streit AG, Koeniz, Switzerland) and placido disk-based topography Topolyzer (WaveLight, Alcon). Predictive IOL power was calculated according to Lenstar with its integrated Barrett formula. On the other hand, for each study eye, the surgeon entered preoperative biometry data into the American Society of Cataract and Refractive Surgery (APACRS) online calculator and calculated the IOL power by using the Modify K values, which, including the individual steep and the flat K values, were decided by the experienced surgeon according to the topography (Nidek OPD-Scan III), AL from Lenstar, and Barrett Universal II formula. These were referred to as Modify method. In all cases during the surgery, intraoperative wavefront refractive biometry was obtained using the ORA (Alcon, Fort Worth, TX, USA) system after cataract extraction and before IOL implantation. From the varying results acquired through Lenstar, Modify, and ORA system, the surgeon used his personal judgment to select the actual IOL power to use.

### IOL implantation

All patients’ surgeries were performed under topical anesthesia, and phacoemulsification was performed through a temporal, clear corneal incision with IOL implantation in the capsular bag. During measurements with ORA, the speculum was carefully adjusted to avoid extra pressure on the eyelid. Aphakic aberrometry measurements were obtained after cataract removal, and the anterior chamber was inflated to a normotensive level (verified between 15 and 21 mm Hg) with Provisc. The measurements were used to immediately calculate the aphakic SE. There were no significant intraoperative complications reported.

### Postoperative outcome measurements

The best-corrected visual acuity and subjective manifest refraction spherical equivalent (MRSE) were obtained in the second month postoperatively. The data were analyzed to determine the real prediction error (PE), absolute PE, and the number and percentage of eyes within a certain refractive PE. The PE was calculated as the difference between the postoperative MRSE and preoperative Lenstar, Modify, and ORA target (predicted) refraction in SE. They were presented as Lenstar target refraction spherical equivalent (TRSE), Modify TRSE, and ORA TRSE.

Several clinical characteristics, including AL, ACD, LT, and the combination of ACD and LT (ACD + 0.5 × LT), were also included to find the differences in PE in the three proposed group. The effective lens position (ELP) was presented by calculating the ACD + 0.5 × LT value obtained by Lenstar preoperatively.

### Statistics

The data on real and absolute PEs were summarized as mean ± standard deviation and median with range. The agreement between the postoperative MRSE and the three proposed methods (Lenstar, ORA, and Modify) was evaluated using the Bland–Altman plot. The PE of target MR was classified into small (≤ ± 0.5 D), medium (0.5 D–1.0 D), and large (> ± 1.0 D) error subgroups. The proportion of subgroups for PE between any two methods (i.e., Lenstar vs. ORA) was compared using the McNemar-Bowker test.

The real PE of target MR for the three proposed methods was further compared using a one-way analysis of variance by different clinical characteristics, including AL (≤ 22 mm, 23–24 mm, and ≥ 25 mm), ACD (≤ 2.9 mm, 3.0–3.3 mm, and ≥ 3.4 mm), LT (≤ 4.3 mm, 4.4–4.6 mm, and ≥ 4.7 mm), and the combination of ACD and LT (ACD + 0.5 × LT: ≤ 5.1 mm, 5.2–5.6 mm, and ≥ 5.7 mm). Finally, Pearson's correlation tested the relationship between preoperative clinical characteristics and postoperative MRSE. All tests were 2-tailed, and *P* < 0.05 was considered statistically significant. Data analyses were conducted using SPSS 25 (IBM SPSS Inc, Chicago, Illinois).

## Conclusion

Intraoperative wavefront aberrometry with the ORA system for IOL power calculation does not provide reliable refraction results compared to the Lenstar and Modify methods in cataract surgery in Asian eyes. A higher rate of more than 0.5 D and 1.0 D residual refraction error was found in ORA than in the other two methods. However, the ORA system demonstrated the best results of IOL power selection in eyes with a normal range of ACD + 0.5 LT (5.2–5.6 mm) in Asian eyes. Further studies are needed to improve the precision and reliability of the measurement in the future.

## Data Availability

The datasets generated during and analyzed during the current study are available from the first author (Hung-Yuan Lin) on reasonable request.
